# ACQ4: an open-source software platform for data acquisition and analysis in neurophysiology research

**DOI:** 10.3389/fninf.2014.00003

**Published:** 2014-01-30

**Authors:** Luke Campagnola, Megan B. Kratz, Paul B. Manis

**Affiliations:** Department of Otolaryngology/Head and Neck Surgery, Curriculum in Neurobiology, University of North Carolina at Chapel HillChapel Hill, NC, USA

**Keywords:** electrophysiology, laser scanning photostimulation, python language, multiphoton microscopy, data acquisition, analysis software, calcium imaging, patch clamp

## Abstract

The complexity of modern neurophysiology experiments requires specialized software to coordinate multiple acquisition devices and analyze the collected data. We have developed ACQ4, an open-source software platform for performing data acquisition and analysis in experimental neurophysiology. This software integrates the tasks of acquiring, managing, and analyzing experimental data. ACQ4 has been used primarily for standard patch-clamp electrophysiology, laser scanning photostimulation, multiphoton microscopy, intrinsic imaging, and calcium imaging. The system is highly modular, which facilitates the addition of new devices and functionality. The modules included with ACQ4 provide for rapid construction of acquisition protocols, live video display, and customizable analysis tools. Position-aware data collection allows automated construction of image mosaics and registration of images with 3-dimensional anatomical atlases. ACQ4 uses free and open-source tools including Python, NumPy/SciPy for numerical computation, PyQt for the user interface, and PyQtGraph for scientific graphics. Supported hardware includes cameras, patch clamp amplifiers, scanning mirrors, lasers, shutters, Pockels cells, motorized stages, and more. ACQ4 is available for download at http://www.acq4.org.

## Introduction

The techniques used in experimental neurophysiology have grown in complexity with the development of new technology. In particular, neuroscientists often combine traditional electrophysiological recordings with multiple types of imaging, photostimulation, and behavioral interactions. These new techniques allow the collection of data with greater resolution, precision, and throughput than ever before. At the same time, they pose new challenges in their technical complexity. Increasingly, experimenters rely on computer automation to orchestrate these experiments and analyze the resulting data. Thus, software is a key component in integrating new technologies into experimental neurophysiology.

We have developed a modular software platform for experimental neurophysiology called ACQ4. The system integrates acquisition, management, and analysis of all experimental data into a single collection of tools. With a few exceptions, ACQ4 was designed as a general-purpose tool and thus we expect it to be of value to the neuroscience community as well as other fields of research.

The primary purpose of ACQ4 is to provide a system to automate experiments combining traditional electrophysiology, photostimulation, and imaging. The broader scope of ACQ4 is to provide a generic system for controlling research equipment including analog and digital input-output boards, cameras, motorized position control, and any devices that can be interfaced through analog or digital channels, serial port communication, or a manufacturer-provided application programming interface (API). ACQ4 is a modular and extensible system, making it possible to add support for new types of devices and experimental protocols. Likewise, ACQ4 makes few assumptions about the hardware configuration, allowing arbitrary combinations and arrangements of experimental hardware. The tools currently included with ACQ4 provide support for a wide range of neurophysiology techniques including patch-clamp electrophysiology, laser scanning photostimulation, calcium imaging, intrinsic imaging, and multiphoton imaging. In addition, the system provides services for data management and an extensible set of analysis tools.

ACQ4 is written in Python and is built entirely on free and open-source tools, with the exception of some commercial hardware drivers. The use of open-source software affords many benefits over closed-source software. First, experimenters with the necessary expertise may modify the program to suit their own needs and contribute the code back to the community for others to use. In this way, open-source software is developed by those who have the best understanding of their own experimental needs. Second, labs need not rely on the survival of a business or a particular product for long-term support. Finally, virtually any task that can be done manually may be automated through the inclusion of custom scripts.

Here, we discuss the design of ACQ4, the supported devices and acquisition paradigms, and illustrate usage of the program in an experimental setting. The software sources and documentation are available at http://www.acq4.org.

## Overview

The design philosophy for ACQ4 was to develop a general-purpose research platform with a strong focus on neurophysiology. In our lab it is currently used for patch-clamp electrophysiology, laser scanning photostimulation, multiphoton microscopy, calcium imaging, and intrinsic imaging. Although ACQ4 has been used most extensively with *in vitro* preparations, it supports a feature set that is broad enough to encompass many *in vivo* preparations as well. The software is capable of handling most aspects of acquiring, managing, and analyzing experimental data.

**Figure 1:**
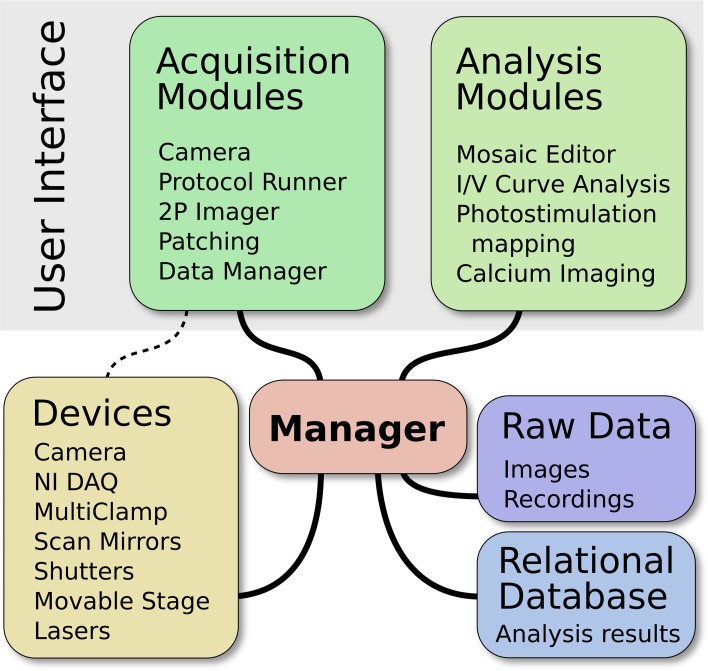
**Architecture of ACQ4**. The central Manager is responsible for configuring devices and facilitating most communication between devices and modules. The user interface is composed of multiple modules, each providing a particular functionality. The Data Manager handles storage, the organization of raw data, and storage of associated metadata.

ACQ4 is both a platform for application development and a suite of modules built on that platform. At the core of ACQ4 is a central Manager that controls access to devices, executes tasks that synchronize the actions of multiple devices, manages the storage and retrieval of data, and loads user interface modules (Figure 1). Each user interface module provides a specific functions such as camera access, synchronized recording and device control, data browsing, and various analysis tasks. These modules make use of the services provided by the Manager, allowing them to communicate with one another.

In most cases, user interface modules control the acquisition hardware indirectly by submitting task requests to the Manager. These task requests specify which devices will participate in the task and describe the intended actions for each device. The manager then handles all aspects of device configuration and synchronization, while ensuring that tasks submitted by different modules do not attempt to access the same hardware simultaneously. This is one of the most important services provided by the Manager because it simplifies the creation of new acquisition modules and at the same time encourages scalability. For situations that require low-level access to the hardware, modules may instead request direct access from the Manager.

The data management system in ACQ4 is designed to emphasize flexibility and longevity. Data is organized into a hierarchy of directories with user-defined structure. Each directory contains a human readable index file that stores annotations and other metadata related to both the directory and any files contained within it. Most data acquired by ACQ4 is stored using the HDF5 file format (www.hdfgroup.org). These files contain both the raw data arrays, for example from camera and digitized recordings, as well as meta-information related to the recordings. ACQ4 provides libraries for reading these files in both Python and MATLAB. When the data is analyzed, the results may be stored in an SQLite relational database file (www.sqlite.org). The use of industry-standard HDF5 and SQLite formats helps to ensure that data is readable by a variety of different applications, both now and in the future.

## Example use cases

The general-purpose nature of ACQ4 makes it impractical to outline all of its possible uses. In addition, its extensibility means that the range of experiments that are possible to perform with ACQ4 will grow as experimenters add functionality. Here we present examples of experiments that are possible with ACQ4 to illustrate a range of functionality.

### Multiphoton calcium imaging during whisker deflection

ACQ4 can be used to perform multiphoton calcium imaging both *in vitro* and *in vivo*. Like most aspects of ACQ4, the pattern of scanning is extremely flexible. For example, an experimenter could perform raster scans over a few dendritic spines and a line scan over a section of dendrite. The position, speed, and frequency of scanning are all definable by the user. This type of experiment would require a laser, a set of scan mirrors, and a signal detection device such as a photomultiplier tube or photodiode.

To record calcium signals during a whisker deflection, an analog output signal is used to drive an actuator to deflect the whisker. At the same time, a video camera records the whisker deflection and exports a TTL signal marking the start time of each frame exposure. All input and output signals are acquired synchronously and the final data generated has multiple calcium imaging components and a whisker video, all with automatically determined timing information.

### Laser scanning photostimulation

Laser scanning photostimulation is a technique used to stimulate a specific, usually small area of tissue (Katz and Dalva, 1994). It involves setting the voltages on a pair of scan mirrors to direct a laser beam to a specific site on the sample, combined with fast gating of the laser beam by a shutter, Pockels cell, or Q-switch. ACQ4 can be used in many types of photostimulation experiments. Some of these include: sequentially stimulating an arbitrary set of sites to search for presynaptic partners to a specific target neuron, using multiphoton stimulation to stimulate an individual spine on a recorded neuron, or stimulating a bundle of axons expressing Channelrhodopsin by performing a line scan across them. Each of these types of experiments require a laser for stimulation, a pair of scan mirrors to set the position of the laser beam, and a camera so that the user can define the laser position relative to the sample. In the first two examples a patch-clamp amplifier is also required for recording from a target neuron.

ACQ4's use of a global coordinate system allows position information to be saved next to recorded data, and for stimulation sites to be automatically reconstructed alongside images of the sample. ACQ4 also allows for the specification of an arbitrary set of stimulation sites, including multiple discontinuous sets of sites.

### *In vitro* patch clamp with drug perfusion

The objective of this example is to record a set of current-voltage relationships before and after drug application from a whole-cell, brain slice preparation. Current-voltage relationships are recorded every minute for three 10 min periods before drug application, during drug application, and after drug washout. In ACQ4, a simple, custom Python script (or a patient experimenter) would control the initiation of a set of I/V curve measurements each minute, and after 10 min activate a digital output line to open a valve controlling the flow of drug solution. After 10 more minutes the script would return the valve to its original position. Importantly, only minimal effort would be required to develop such a script because execution of the I/V curve would only involve recalling a task definition that was previously designed through a graphical user interface. Devices required for this type of experiment would include a patch-clamp amplifier and valve that could be easily defined as a generic digital device (see below, Generic analog/digital devices).

### *In vivo* recording during an operant conditioning task

In this example, the objective is to record multi-unit activity in premotor cortex during an operant conditioning task with auditory discrimination. This type of task might include 4 analog inputs from a tetrode array, a digital output that controls an LED serving as a cue to the animal that a trial has started, an analog output that controls the auditory stimulus, a digital input that reports a lever press, and a digital output that controls a valve to release a reward.

The most complex aspect of this experiment is orchestrating the simultaneous tetrode recording, LED cue, auditory stimulus, and lever press input. However, this task can be defined quickly and interactively by the user in ACQ4. The remainder of the experiment—analyzing the behavioral response and administering the reward, would then be handled by a custom script.

## Related projects

ACQ4 is designed as a platform encompassing a wide range of acquisition capabilities as well as data management and analysis. We developed this software initially to address a few specific, *in vitro* experimental paradigms. However, in covering that range of experiments we have devised a generalized architecture which can be applied to many common experimental paradigms. Although no other projects provide the same breadth of features, several are actively developed that have overlap with parts of ACQ4 or provide features that are not currently available or practical in ACQ4.

Of the software systems providing similar acquisition features to ACQ4, most are closed-source and sold commercially. The most notable exception is Ephus (Suter, 2010), an open-source system based on the commercial platform MATLAB that was developed to combine electrophysiology and laser photostimulation in a modular, general-purpose acquisition system. Ephus was developed alongside ScanImage (Pologruto et al., 2003), which supports laser scanning microscopy. The features provided by the combination of Ephus and ScanImage overlap significantly with those of ACQ4. However, ACQ4 integrates all of these functions as modular components in a single application, whereas Ephus and ScanImage are implemented as separate applications. There are advantages and disadvantages to both approaches, although we find the approach in ACQ4 to be more flexible in that it allows stronger integration between modules, and additionally may be operated as multiple, independent processes if desired. Another open-source program with overlapping functionality is WinWCP (http://spider.science.strath.ac.uk/sipbs/software_ses.htm), which is a Windows-based acquisition application with a strong focus on whole-cell recording techniques.

MANTA (Englitz et al., 2013) and NeuroRighter (Rolston et al., 2010) are open-source acquisition systems designed for use with multi-electrode arrays and are based on MATLAB and Microsoft.NET, respectively. Both projects provide features that fall within the *intended* scope of ACQ4, but are not currently implemented in a practical or optimized way. MANTA is optimized for use with large arrays containing over 100 channels, whereas ACQ4 has been used mainly with experiments involving a small number of electrodes and would require new user interface development to make the use of large electrode arrays practical. Similarly, NeuroRighter was designed to allow low latency, closed-loop feedback in multichannel electrophysiology, whereas ACQ4 has only been used for open-loop recording and stimulation. Both of these are desirable areas for future development in ACQ4. Another notable project is Open Ephys (http://open-ephys.org), which has developed both open-source hardware and software for electrophysiology and optogenetics applications. Like MANTA and NeuroRighter, it has a strong focus on multichannel recording.

Several projects exist that aim to provide either standard data formats for biological data or platforms for sharing and collaborating such data. BioSig (Vidaurre et al., 2011), Neo (http://neuralensemble.org/neo/), EDF+ (Kemp and Olivan, 2003), NeuroShare (http://neuroshare.org), and Pandora (http://software.incf.org/software/pandora) are all data standards and interoperability projects in various states of development. Although ACQ4 uses its own internal data format, it is desirable to have the ability to export data from ACQ4 to these standardized formats. Likewise, projects such as CARMEN (Austin et al., 2011), CRCNS (http://crcns.org/), INCF (http://www.incf.org), and Brainliner (http://brainliner.jp) provide platforms for data sharing and collaboration, and thus would be useful to integrate with ACQ4. Numerous open-source analysis projects such as OpenElectrophy (Garcia and Fourcaud-Trocmé, 2009), Spyke Viewer (Pröpper and Obermayer, 2013), and Stimfit (Schlögl et al., 2013) also provide features that are largely complementary to the analysis features in ACQ4. Support for projects like Neo may facilitate data transfer between ACQ4 and external analysis tools such as these.

## Program details

### Platform

ACQ4 is written in Python, a modern, open-source programming language that has grown rapidly to become one of the most popular general-purpose programming languages in use. Python is also unencumbered by commercial licensing, which greatly reduces barriers for other researchers to access ACQ4. As an interpreted language, Python is easier to use than most compiled languages at the cost of being less efficient. However, ACQ4 is able to achieve high performance by using optimized libraries that provide efficient numerical processing (NumPy and SciPy; http://www.scipy.org), user interface (PyQt; http://www.riverbankcomputing.com), and scientific graphics (PyQtGraph; http://www.pyqtgraph.org).

The combination of these components—Python, NumPy, PyQt, and PyQtGraph—allows ACQ4 to operate efficiently on most platforms using entirely free software. ACQ4 currently runs on Windows, Linux, and OSX. However, the availability of hardware drivers may restrict the platforms available for data acquisition. For example, the cameras that are currently supported only provide Windows drivers and MultiClamp amplifiers (Molecular Devices) do not support Linux. However, data display and analysis work on all supported platforms.

### Device management

Each type of device supported in ACQ4 is represented internally as a Python class that inherits from a base Device class. Adding support for a new type of device requires writing a new Device subclass. Four generic Device subclasses (described below) provide common functionality across groups of similar devices: DAQGeneric, Camera, Stage, and Laser. These generic classes reduce code replication and help encourage all compatible devices to adopt a similar interface. At a minimum, each Device subclass defines the set of configuration options it accepts and methods for interacting with the hardware. Although this minimal interface is sufficient to define a Device class, most devices will also implement one or more high-level interfaces that allow it to interact with other parts of ACQ4.

The Manager keeps track of all devices in the system as defined in a human-readable (text) configuration file. This file assigns unique names and specifies the type of each device, in addition to any information needed to open the device, such as serial port numbers or other device-specific identifiers. The configuration file also specifies the relationships between devices. This includes both electrical connections (for example, the output of an amplifier is connected to a specific analog input channel of another device) and physical relationships (for example, a microscope is rigidly connected in some orientation to a movable stage). When ACQ4 is started, the Manager attempts to establish and maintain a connection with each defined device. The Manager then presents a centralized interface providing three main features: a general architecture for managing a system of interconnected devices from which new acquisition applications may be built; device reservation, allowing modules to operate independently of one another without concern for resource collisions; and hardware abstraction, allowing modules to transparently support multiple types of devices having the same base class.

Device subclasses will usually communicate with their hardware either through a serial port, through a manufacturer-provided C API, or through analog/digital channels on an acquisition board (or by some combination of those methods). For serial communication, ACQ4 provides a simplified interface built on the PySerial package (http://pyserial.sourceforge.net/). For access to C APIs, ACQ4 uses Python's built-in ctypes package in addition to a system for parsing C header files that automatically extracts the needed function signatures and constant values. For communication via analog/digital ports on an acquisition board, ACQ4 includes a Python wrapper for the National Instruments DAQmx library.

### Optomechanical devices

The experiments that ACQ4 is designed to handle often involve multiple devices whose spatial relationships to each other must be calibrated, tracked, and reported. For example, a user may wish to collect a set of images from a range of locations across a sample, mark locations for later reference, or direct a scanning laser to specific sites in the sample. To accomplish this, a global coordinate system is used throughout ACQ4 to represent the physical coordinates of the sample. Any recording or stimulation that has a defined spatial relationship to the sample is automatically registered with the global coordinate system. Thus, images and photostimulation data are automatically stored alongside their global position and scale, allowing automatic reconstruction of image mosaics (multiple tiled images).

A broad subclass of devices, referred to as optomechanical devices, represent hierarchically-linked hardware with defined physical or optical relationships to one another and, ultimately, to the global coordinate system. The choice of an appropriate global coordinate system is arbitrary and left to the experimenter, although in systems which use any type of imaging, the global coordinate system is typically chosen to be fixed relative to the imaged subject. Static relationships between devices are specified in the device configuration file, whereas any changes in dynamic relationships (for example, when a motorized stage moves, or an objective lens is changed) will be immediately reflected in the coordinate system transformations between devices in the hierarchy. In most cases, the static configuration is determined and written manually. For more complex relationships, however, automated calibration functions may be used to assist in generating the necessary configuration.

**Figure 2:**
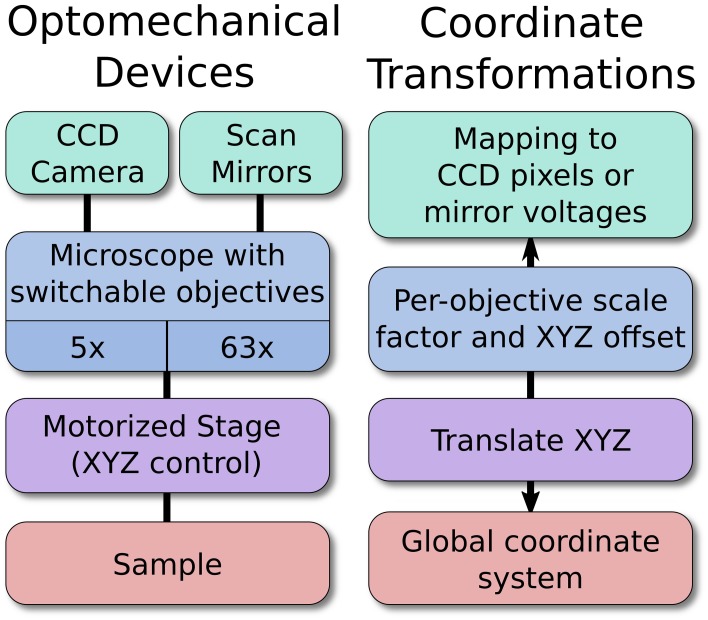
**An example hierarchy of optomechanical devices**. The optomechanical device arrangement allows the software to map specific locations on a sample to a pixel on the CCD, or to the proper pairs of scan mirror voltages. The position of the stage, objective magnification, and per-objective offset are all handled automatically. New devices may be added anywhere in the hierarchy, which allows ACQ4 to support arbitrary hardware configurations.

For example, a motorized stage, microscope, and camera may all be linked optomechanical devices (Figure 2). As the stage moves, the global coordinate location of the microscope and camera will shift to reflect this new arrangement. Likewise, changing the objective lens currently in use will change the optical scaling and offset associated with the microscope, which in turn defines the boundaries of the camera sensor relative to the sample. In this example, the scaling of the camera sensor coordinates would be measured manually under different objective lenses by imaging a calibration target or by moving the sample by a known distance. Because all coordinates are represented in 3D, it is also possible to seamlessly and transparently add Z-control such as a motorized focusing mechanism.

The end result is that devices in the optomechanical hierarchy generate data that is registered to the physical coordinates of the sample, and this requires no effort from the user during the experiment as long as ACQ4 is able to record positioning information. The structure of this device hierarchy is entirely user-definable, allowing ACQ4 to work with arbitrary device configurations.

### High-level synchronized device control

Although it is possible to directly interact with each device, the Manager also provides a high-level system that handles all details of configuring and synchronizing devices to perform complex acquisition tasks. User interface modules may acquire data by submitting task requests to the Manager, which runs each queued task in order as hardware becomes available. This system greatly reduces the effort required to develop new data acquisition modules by providing a simple and flexible language for describing a set of synchronous device activities.

Despite its flexibility, the task execution system cannot handle all possible use cases. Notably, it does not automatically handle task repetition or sequences. Instead, this functionality is implemented separately in the user interface modules to ensure that the task execution system stays as simple as possible. Another limitation is that the task execution system only supports short (episodic) acquisitions. Continuous acquisition is currently under development and will greatly expand the range of capabilities supported by the task system. Further limitations may exist for each type of device, depending on the range of features it exposes to the task execution system. Nevertheless, modules which require low-level access to their devices may do so directly and are only required to make use of the Manager's device reservation system, ensuring both flexibility and scalability.

#### Task specification

Task specifications consist of a set of high-level commands describing the desired behavior of each device participating in the task. Each device defines the exact set of commands it will accept; typically these include stimulus waveforms, the names of channels to record from, triggering settings, and other device-specific options. Internally, a task is specified as a Python dictionary with one key per device and a “protocol” key which specifies general options applying to the entire task. The following example task specification instructs the Manager to record 100 ms of data simultaneously from a current clamp amplifier and a camera, with the camera triggering the start of the recording:

protocol:
   storeData: False
   continuous: False
   duration: 0.1
DAQ:
   rate: 40e3
   numPts: 4e3
   triggerDevice: Camera
   # DAQ waits for trigger from camera
Clamp1:
   mode: 'I=0'
   primary:
      mode: 'MembranePotential'
      record: True
Camera:
   record: True
   triggerMode: 'Normal'
   # camera does not wait for trigger
   channels:
      exposure:
         record: True
         # record frame exposure times


In the above Example, three separate devices must be configured to operate synchronously: a data acquisition device (DAQ), a single current clamp channel (Clamp1), and a camera. The names of these devices correspond to those defined in the device configuration file, which also includes information about the connections between devices. For example, the primary output of the MultiClamp channel is connected to a particular analog input on the DAQ, and likewise a TTL start signal from the camera is connected to a particular digital input on the DAQ from which it can trigger the start of the acquisition. Because the relationships between devices are stored in a local configuration file, it is not necessary to specify the relationships in task definitions. Thus, a single task definition may function across multiple hardware configurations only by changing the device names it references.

Note that, in executing a task, the Manager only uses information in the “protocol” section of the task structure; the commands provided for each device must be interpreted and acted upon by the devices themselves. In this way, ACQ4 ensures flexibility in the task execution system by allowing new types of devices to define the command structures that best suit them. For a device to be compatible with the task execution system, it must implement a specific interface through which it will communicate with the Manager. The details of this interface are discussed in the ACQ4 developer documentation.

Task specifications such as the example above are generated by user interface modules based on some input provided by the user. In most cases, these modules provide a streamlined graphical interface for controlling a particular type of acquisition; for example, the Imager module performs scanning laser microscopy by submitting task requests that include commands for scanning mirrors, laser power control, and photomultiplier readout. At the opposite extreme, the TaskRunner module provides a graphical system for designing nearly arbitrary tasks and viewing the results. ACQ4 facilitates the development of new modules by abstracting the complexities of device programming into a simple, high-level task specification.

#### Task execution

After a module has generated a task structure, it submits the task to the Manager, which then executes a series of steps to ensure that all devices are configured properly and started in the correct order. First, the Manager waits until all requested hardware has become available. This ensures that tasks are handled in a thread-safe manner, allowing modules to request and wait on tasks in a background thread while the main thread displays results and processes user input. Next, the Manager iterates over all of the devices in the task, asking each whether it has a preference for being configured before any others. Devices are then configured in an order that both satisfies the ordering preferences and minimizes the time until the task may be started. For example, some cameras have a long delay period for arming their external triggers; such devices are configured as early as possible while still obeying the required ordering constraints. The details of the configuration stage differ depending on the devices in the task, but typically involve sending stimulus and triggering waveforms to the data acquisition board, preparing devices to receive triggers, and configuring device-specific settings such as gain values or sample rates. After configuration, the Manager asks each device whether it should be started before or after any other device, based on the specified triggering options. This allows, for example, a camera to be triggered by a digital output in one task, or for the data acquisition hardware to be triggered by the same camera in another task. Finally, each device is started in the correct order. During this stage, any device may delay starting until its hardware is ready to begin the task.

At this point, control returns to the module that requested the task. The module may then either poll to determine when the task has completed, or abort the task immediately. If all devices in the task indicate that they have successfully completed their task (the criteria for completion are different for each device), then the module may request the results of the task execution. These results are packaged as a Python dictionary with one key per device, and the format of each value is determined by the device itself. Modules may then display the results or perform online analysis. If requested, the task results may be automatically stored to disk for later retrieval and analysis. The following example outlines a typical task execution:

# Create a task from the previously
  generated taskStructure
task = manager.createTask(taskStructure)

# Start the task
task.execute()

# While waiting for the task to complete,
  process user events
# and check whether an abort was requested
while not task.isDone():
   processUserInput()
   if userRequestedAbort:
      task.stop(abort=True)
      raise Exception("User aborted
       acquisition.")
# Finally, get the results
results = task.getResults()


In this example, the taskStructure variable would contain a structure similar to the task specification described in section Task Specification.

### Supported hardware

ACQ4 supports a range of hardware devices for data acquisition and control. Most of these devices fall under one of five categories: data acquisition boards, analog/digital signal devices, cameras, lasers, and position control devices. Each device is implemented as a subclass of the base Device class or one of the generic Device subclasses (DAQGeneric, Camera, Laser, or Stage). New device support may be added by creating new Device subclasses.

ACQ4 currently defines four programming interfaces that a device may optionally implement to take advantage of the high-level services offered by the Manager and user interface modules. First, devices may implement the task execution interface, allowing them to be controlled by the task execution system described above. Second, a TaskRunner interface provides a control panel allowing the user to graphically specify the desired behavior of a device when it is included in a TaskRunner module (described below). This interface is responsible for generating device-specific task commands based on user input and for communicating any user-defined sequence parameters to the TaskRunner. Third, devices may implement a graphical control panel which allows immediate control and monitoring of the state of the device, independent of any tasks being run. This is used, for example, to manually open and close shutters, configure amplifier modes and camera settings, and to initiate calibration routines. Finally, a Camera interface provides a control panel for display in the Camera module in addition to methods allowing the module to start and stop the camera. The details of these interfaces are discussed in the ACQ4 developer documentation.

#### Data acquisition boards

Data acquisition boards are commonly the central hub of data acquisition and device control. They are responsible for recording analog signals from physiology equipment, synchronizing cameras, driving galvanometric scan mirrors, and communicating with a variety of other hardware such as LEDs, shutters, Pockels cells, photodiodes, and Q-switched lasers. ACQ4 supports National Instruments devices via the DAQmx library and has been tested with E- and M-series data acquisition devices, although other series may work as well.

Support for these devices is provided by the NiDAQ Device subclass, which implements both the task execution and TaskRunner interfaces. Together, these interfaces allow simple configuration of the sample rate and number of samples to acquire during a task. Additionally, this device may be triggered by a TTL signal from any other device with a defined connection to a PFI digital port.

Currently, no other data acquisition devices are supported in ACQ4. The development of a generic Device subclass for data acquisition boards, and for support for similar devices such as Digidata and InstruTech ITC, are desirable future developments.

#### Generic analog/digital devices

Although data acquisition boards are one of the most important hardware components used in ACQ4, the user rarely interacts directly with these devices. Instead, each device that is connected to analog or digital channels on the board is responsible for communicating with the NiDAQ Device to configure the appropriate channels and triggering settings. The user specifies actions, waveforms, and recording criteria through the user interfaces implemented by each device class. For example, to perform LED stimulation in an optogenetic preparation, the user would interact with the LED device in ACQ4, and this device would subsequently transfer its stimulation waveforms to the NiDAQ device it is connected to. The benefits to this approach are that each device is free to define the user interface that is most naturally suited to that particular device, and that users are unconcerned with the details of the data acquisition board.

Most devices that are controlled by analog or digital channels are defined as subclasses of the DAQGeneric class. This class simply represents a logical grouping of analog and digital ports, such as the set of input and output ports connected to an amplifier, or the digital lines from a camera. DAQGeneric implements the task execution, TaskRunner, and device control interfaces described previously. The TaskRunner interface provides a plot display area for each channel and a simple function generator control for output channels. Due to the highly generic nature of this class, it is common for its subclasses to reimplement substantial portions of its behavior, usually to provide a user interface that more intuitively reflects the capabilities of each device. For example, Cameras are subclasses of DAQGeneric which provide imaging functionality that is complemented by the ability to record a camera's TTL exposure signal, or to generate triggering waveforms to drive the camera exposure.

#### Cameras

Support for scientific cameras currently includes all devices which use either PVCam (Photometrics) or QCam (Q-Imaging) drivers. Cameras support live-imaging modes as well as controlled data acquisition modes that specify the timing and behavior of the device. In live-imaging mode, the camera collects frames freely and sends them to a user-interface module for display. This mode is generally used for visualizing the preparation throughout the experiment including while navigating and during placement of electrodes for stimulating or patching. Cameras may also make use of connections to data acquisition channels. During task execution, the camera may be triggered by the data acquisition board or serve as the starting trigger for another device.

In addition, many cameras export a TTL signal that indicates the timing of frame exposures. When it is recorded, this signal is analyzed to determine starting exposure time of each camera frame, allowing the precise synchronization of imaging and electrophysiology or other signals. Image data is stored to disk alongside the raw exposure and trigger signals, and the time values of each frame are stored as meta-data. The result is that physiological recordings made synchronously with imaging can be automatically registered temporally during analysis.

Cameras are treated by ACQ4 as optomechanical devices, and thus may be calibrated such that their size, position, and orientation have a fixed spatial relationship to any other optomechanical devices. This is most commonly used with both a motorized stage for position feedback and a microscope device which defines per-objective scaling and offset. With a properly configured system, image mosaics can be collected and automatically reconstructed.

#### Electrophysiology amplifiers

Three amplifiers are currently supported for electrophysiology experiments: the MultiClamp 700A and 700B, and the AxoPatch 200. In addition, any device lacking computer control (for example, the AxoProbe 1A) may be used as a generic analog device.

ACQ4 records all remotely accessible parameters from the MultiClamp Commander software (Molecular Devices) or from the analog telegraph outputs on the AxoPatch 200. For the MultiClamp, several parameters such as the VC/CC mode, gain, and signal selection may also be controlled from the user interface. ACQ4 automatically applies the appropriate scaling constants for input and output to the analog channels.

Switching between voltage and current clamp is handled automatically by the device, first switching to *I* = 0 mode before changing the holding commands sent to the analog output. This allows ACQ4 to rapidly and safely switch between recording modes without user interaction. The AxoPatch 200 lacks computerized control; in this case ACQ4 prompts the user to switch modes when necessary.

#### Scan mirrors

Galvanometric scan mirrors (scanners) are commonly used as laser steering devices for both multiphoton imaging and photostimulation. ACQ4 supports these devices by exporting two calibrated analog output signals via a data acquisition board. For experiments combining laser scanning and patch-clamp recording, it is necessary to have at least three analog output channels available.

ACQ4 scanner devices allow the user to graphically specify the desired location of a laser beam in the global coordinate system. Since ACQ4 considers scanners to be optomechanical devices (see Optomechanical devices above), they are aware of both their position relative to the sample and the details of any optics in between. The primary job of the ACQ4 scanner device is to perform a basic coordinate mapping: given an x, y location in the global coordinate system, determine the voltages V_*x*_, V_*y*_ that will cause a specific laser (multiple lasers may share the same set of mirrors) to be centered on the global location specified.

This mapping is determined by an automated calibration system that uses a camera to record the laser spot location while scanning over a range of mirror voltages. From this data, the coefficients of a generic mapping equation are solved using a Levenberg–Marquardt least-squares optimization (scipy.optimize.leastsq):

$$\begin{array}{l} V_{x}=Ax^{2}+By^{2}+Cx+Dy+E\\ V_{y}=Fx^{2}+Gy^{2}+Hx+Iy+J \end{array}$$

Some systems use multiple objective lenses or multiple lasers which enter the microscope from different angles, so the mapping coefficients are determined once for each combination of source laser and microscope objective.

Scanner devices support three modes of operation during task execution. For simple photostimulation experiments, the output voltage is set immediately before each task run. For more complex scanning patterns such as with multiple sequential photostimulation sites or arbitrary scanning imaging patterns, a simple command language can be used to specify raster, line scan, circular, and spiral scan shapes. This mode is sufficient for modestly complex photostimulation and imaging experiments. For any use not supported by the simple command language, arrays of x, y locations may be specified instead.

#### Lasers

ACQ4 supports calibrated laser output using Pockels cells, shutters, and Q-switched lasers. Laser devices report their current output power either using a beam sampler and calibrated photodiode, or via the power reporting built in to Coherent laser systems. The laser device also keeps a list of calibrated attenuation factors (one per optical train configuration) that are determined by measuring the beam power at the sample plane. By combining the current output power and the correct attenuation factor, the device determines the expected power at the sample. This estimate can be used to allow the user to specify the amount of energy to deliver during short photostimulation flashes by adjusting the pulse duration. A Pockels cell device currently under development will further allow calibrated, analog control of the laser power level over longer scanning tasks. In particular, this will be used to allow laser scanning imaging procedures to be carried out with a well-defined power level to avoid damaging the sample. By accounting for the current output power of the laser, we are able to ensure repeatable power delivery over long-term experiments where it is expected that the output power of the laser may drift, or optical components may change.

#### Stages

Due to the spatial awareness that is built in to many aspects of ACQ4, it is recommended to use a stage or movable microscope objective with some sort of position feedback. ACQ4 has support for the Sutter MPC200 controller which is frequently used in motorized stage systems. The MP285 is also supported with the use of custom interfacing hardware described in the ACQ4 documentation.

Stages are represented in ACQ4 as optomechanical devices whose offset is determined by the position reported by the stage feedback. In the hierarchy of optomechanical devices, stages are typically the highest-level parent device. Thus, stage motion is immediately propagated to all devices which are optomechanically linked to the stage. This allows imaging data to be recorded alongside the location of the image, which facilitates the automated reconstruction of image mosaics. The Camera user interface module, described below, has several features which make use of this optomechanical transformation information. Stages also support motorized position control, which is used by the Imager module to acquire tiled image mosaics and Z-stacks.

#### Microscopes

Microscope devices are a special type of optomechanical device that account for coordinate system changes caused by switching objective lenses. Microscope device configuration files statically define a set of objective slots and a set of objective lenses that may appear in each slot. This allows the experimenter to indicate what type of lens currently occupies each slot, and which slot is currently in use. The currently active slot may also be determined automatically by a Switch device. For microscopes with only two objectives, the switch device may simply correspond to a digital input on the data acquisition board. Then, a microswitch or optical switch attached properly to the microscope can automatically determine the currently active objective.

Each objective defines a unique name, a position offset, and a scale factor. This information is used to ensure that images taken under different objectives are properly aligned and scaled relative to each other. The Camera module reads information about the current state of all optomechanical devices when it writes image files to disk. Thus, all stored images are tagged with the name of the currently active objective. State switches in the optomechanical device hierarchy are also used to trigger some optical devices to use a different calibration. For example, scan mirrors may have different calibrations for different objectives, and lasers may use per-objective attenuation factors when estimating the power at the sample.

### User interface modules

ACQ4's user interface is divided into modules, each providing support for a specific type of activity such as displaying a camera feed, designing and running a task, patching a cell, or browsing through data. Modules are opened via the Manager, and each module is contained in its own window. Development of modules is one of the primary ways that ACQ4 may be extended to perform new functions.

#### Camera module

**Figure 3:**
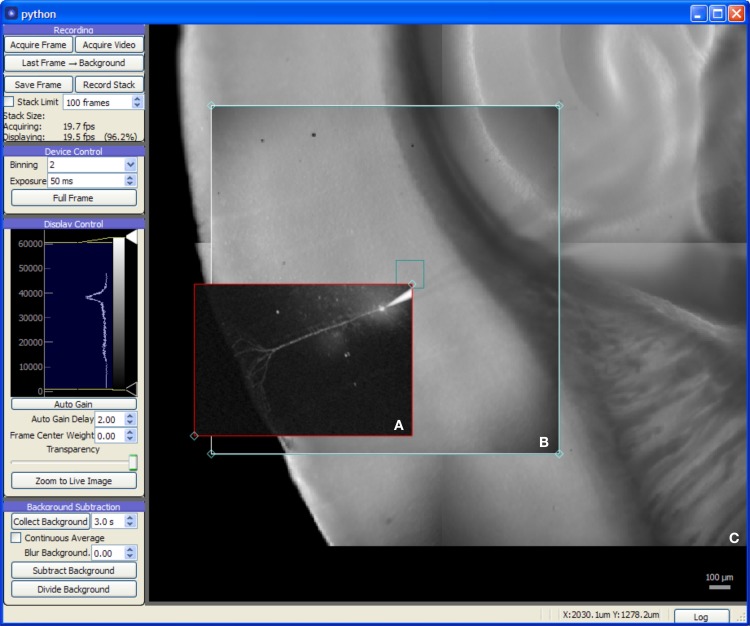
**Screen capture of the Camera module during a mouse auditory cortex brain slice experiment**. All experimental procedures were approved by the Institutional Animal Care and Use Committee at the University of North Carolina at Chapel Hill. The acquisition system consists of an NI-6259 data acquisition board, a MultiClamp 700A amplifier, a Photometrics QuantEM 512SC camera, and a custom multiphoton imaging system (Cambridge Technologies 6510H galvanometric scan mirrors, Coherent Chameleon Vision II ultrafast Ti:Sapphire laser, and a Hamamatsu H7422P-40 photomultiplier incorporated onto a modified Zeiss Axioskop 2 FS microscope with 5× and 63× objectives). The recording chamber was mounted on a 3-axis motorized stage (Mike's Machine Co., Boston, MA) driven by a Sutter MP-285 controller (however, use of the MPC-200 instead of the MP-285 is strongly preferred). **(A)** 2-photon image of fluorescence from an Alexa Fluor 568 labeled cortical neuron recorded in whole-cell tight seal mode. The red rectangle is a draggable region defining the area to be imaged. **(B)** Live video from CCD camera. **(C)** Background frames previously acquired with camera provided a wide-field, persistent view of the brain slice.

The Camera module (Figure 3) provides a live video feed from one or more imaging devices and also serves as a visual representation of the global coordinate system for photostimulation and laser imaging controls. It may be used to record single frames or to stream video to disk alongside any available metadata including the imaging device settings in use, optical train state (such as which objective lens is in use), and the coordinate transformation needed to determine the original location and scale of the image.

The Camera module also provides image processing features helpful for enhancing image contrast for cell patching and calcium imaging. For patching, the module's background subtraction features remove optical artifacts and provide enhanced contrast by flattening the illumination gradient across the image. For experiments using calcium imaging (or other fluorescent indicators), the module provides background subtraction with a continuously-updating background. This acts as a high-pass filter, allowing active fluorescence signals to be quickly identified. Additionally, the module will plot the intensity over time of a region of interest, allowing a local fluorescence signal to be monitored by the experimenter.

The display area of the Camera module serves as a visual representation of the global coordinate space. In addition to images generated by the camera, it also displays data and user interface controls generated by other modules. A module for laser scanning imaging, for example, displays a rectangle in the Camera module window that can be positioned by the user to determine the area imaged with a multiphoton laser system. Likewise, photostimulation sites, grids, and scanning paths are all displayed in the Camera module allowing photostimulation patterns to be defined in relation to either camera or multiphoton imagery. Online analysis modules may also use this space to display photostimulation mapping results or images generated in scanning imaging tasks.

On systems with access to stage position information, the Camera module will track the current stage position and update the virtual coordinate space accordingly. This allows, for example, marking the location of specific cells or sites in the preparation, configuring large, multi-part photostimulation maps, and building large image mosaics which are displayed behind the camera feed. For systems with multiple microscope objectives, the ability to build such mosaics provides a persistent, low-magnification view of the sample, allowing the experimenter to navigate around the sample under high magnification without switching objectives. Likewise, photostimulation patterns may be arranged over large, arbitrary areas outside the visible range of the camera sensor.

#### Taskrunner module

**Figure 4:**
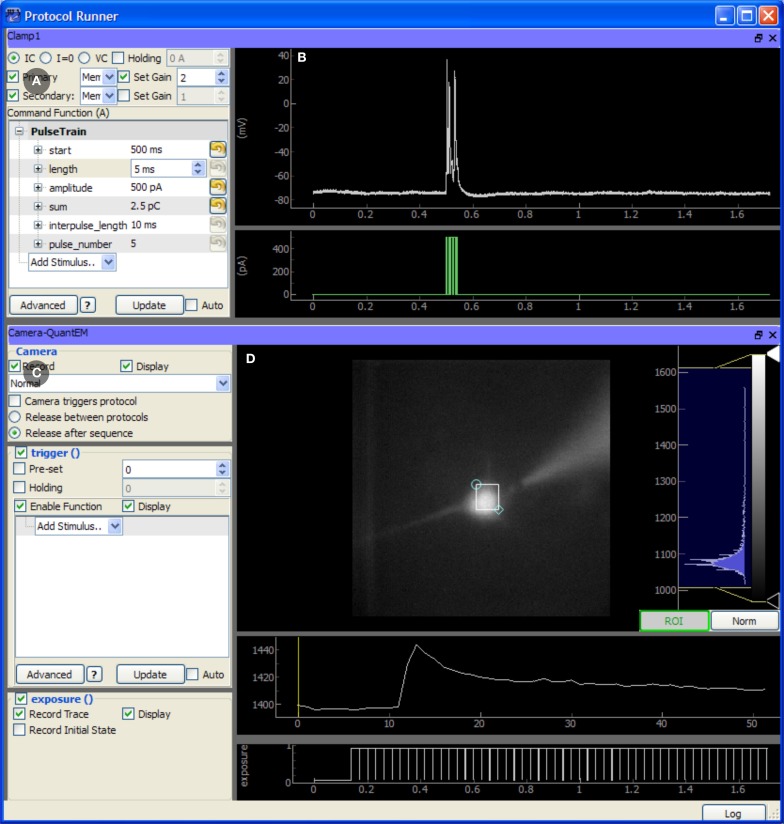
**Screen capture of the TaskRunner module running a calcium imaging task during recordings from an mouse auditory cortex neuron in a brain slice experiment**. The acquisition system is described in Figure 3. The cortical neuron is filled with the calcium indicator Fluo-4 (200 micromolar) and is electrically stimulated through the patch pipette. Additional control panels for selecting devices, running protocol sequences, and configuring the data acquisition board are hidden. To design this task, the experimenter has already selected the camera and patch-clamp channel to be included, and has rearranged the panels to optimize use of the window space. The task has been executed once, and the results are displayed in the rightmost panels. **(A)** Control panel for configuring the behavior of the patch clamp amplifier, including the output waveform specification. **(B)** Plot showing the most recent electrode recording and the command waveform. **(C)** Interface for controlling the Camera. This includes control over the camera's frame transfer mode and triggering waveform. **(D)** The recorded video data is displayed and a region of interest defines the pixels that are averaged together and plotted in the traces below, showing the calcium transient evoked by action potentials in the cell. The bottom-most plot shows the exposure times of acquired camera frames for reference to the electrical recording.

The TaskRunner is a customizable interface to ACQ4's task execution capabilities (Figure 4) and is where most of the user interaction during data acquisition occurs. It allows the user to interactively design and execute a wide range of multi-device tasks including basic patch-clamp stimulation and recording, laser scanning imaging and photostimulation, synchronized multichannel recordings, and complex stimulation paradigms. In this context, the word “task” is used to describe a single, coordinated action to be taken by multiple devices in synchrony. Typically a task involves a short period of recording from electrode amplifiers, cameras, photomultiplier tubes, and other analog or digital channel devices. Arbitrary waveforms may be defined to control stimulation devices, scanning mirrors, and triggering behavior.

When designing a task, the user first selects the subset of devices which should participate in the task. Once selected, each device displays a control panel inside the TaskRunner window, and the user may interactively resize, rearrange, and stack each of these panels as needed. Each control panel provides a set of controls for determining how the device will behave during the task, and a set of display areas where the acquired data for that device will be shown. Synchronization between devices is achieved by specifying the control and triggering waveforms used by each device, while the Manager and data acquisition hardware ensure that tasks are executed correctly. Waveforms are specified either by combining predefined elements such as square pulses or by evaluating an arbitrary Python expression that generates the output array.

The TaskRunner module also allows the execution of sequences of equal-duration tasks which iterate over multi-dimensional parameter spaces. Typically, variable parameters affect some aspect of a stimulus waveform such as the starting time or amplitude of a pulse. However, each type of device defines the ways in which it can sequence variable parameters. Scanning mirror devices, for example, may define an arbitrary set of photostimulation locations, allowing the same task to be executed once for each location. If multiple sequencing variables are specified, the module executes the task once for each point in the multi-dimensional parameter space. This allows the parametric exploration of arbitrary stimulus spaces in a relatively unrestricted fashion, limited only by the recording time and the size of the data set. Optimized and adaptive parameter space searches are currently not implemented, but are a desirable feature for future development.

#### Data manager module

The Data Manager module allows the user to browse, view, and export data, view experiment logs, and manage annotations and other meta-data. Additionally, this module is used to specify the default storage location for data during an experiment. To streamline experiment execution, all modules record data into this default directory rather than prompting the user for a location.

For large studies, keeping data properly annotated and organized consistently is both essential and time consuming. The Data Manager encourages consistent, hierarchical organization of data by allowing the user to define a set of directory types, each having its own set of meta-data fields. These fields may be configured by the user at the beginning of a series of experiments to encourage the user to store and annotate data with a consistent organization. During an experiment, the user simply indicates key transitions such as placing a new sample on the microscope or patching a new cell. The data manager uses these transitions to construct a hierarchy of directories which organize the experimental data and prompt the user to supply the necessary meta-data.

#### Other modules

**Figure 5:**
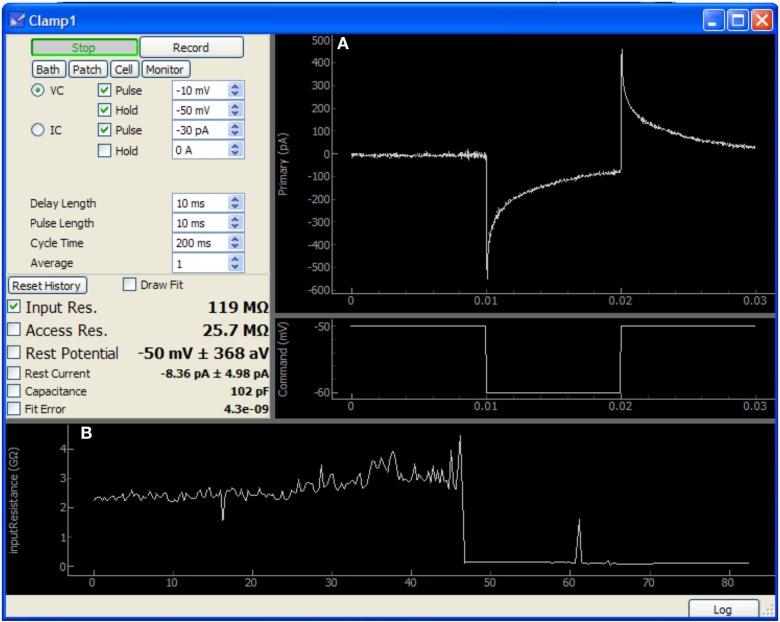
**Screen capture of the Patch module after patching a neuron in a mouse cochlear nucleus brain slice**. In this image, approximately 40 s have passed since the membrane was ruptured to begin whole-cell recording. This data was acquired using only an NI-6259 acquisition board and a MultiClamp 700A amplifier. **(A)** Plots showing the voltage clamp recording and command waveform. This data is used by the module to determine the membrane properties shown at center-left (input resistance, access resistance, etc.). **(B)** Plot showing history of input resistance over the last 80 s. Maximum seal resistance and time of break-in are both recorded in this data.

Several included modules provide more focused features such as scanning laser imaging, assisted cell patching (Figure 5), cell health monitoring, direct device control, and an interactive Python prompt. For features not provided by the core set of modules, ACQ4 is designed to be extensible by allowing the development of modules to support new types of data acquisition and analysis.

The Patch module sends a simple test pulse to the patch amplifier in order to measure properties of the electrode or cell. It is configured to rapidly update during patching and displays the input and access resistances calculated from the response to the test pulse. After patching is complete, this module may be run periodically in the background to monitor cell health. Resistances, holding current, resting potential, and capacitance are all collected and stored as a record of the patching procedure and cell health throughout the duration of the experiment. These values may also be plotted to assist the experimenter in tracking changes over time.

The Imager module provides laser scanning imaging functionality for multiphoton and confocal microscopes. This module combines control of scanning mirrors, laser power, and signal detection devices (such as photomultiplier tubes or photodiodes). Like other modules, the Imager module operates in the global coordinate system and displays its output in the display area of a Camera module. It also displays a user-positionable rectangle which defines the extents of the laser scanning area. The Imager module supports overscanning to remove retrace artifacts as well as bidirectional scanning with automated field shifting to reduce comb artifacts. While the interface includes detailed control of scanning parameters, tiling, and the collection of image stacks, common functionality such as fast (video) scanning or the collection of standardized high-resolution images may also be accessed from preset configurations to simplify user interaction during experiments.

### Analysis modules

Whereas it is possible for a generic acquisition system to cover a wide variety of experimental needs, data analysis must often be tailored to each specific problem. ACQ4 does not attempt to solve all analysis problems. Instead, it facilitates analysis through three approaches: First, complete analysis tools are provided for the most common tasks. Second, ACQ4 implements a modular analysis framework that encourages the development of simple, reusable, and recombinable components. Last, ACQ4 facilitates the use of external analysis applications by storing data in the standard HDF5 format and likewise by allowing export to other formats such as CSV and TIF. Future development may allow export to other standard formats.

Analysis modules in ACQ4 consist, in their most basic form, of a single function which computes a simple analysis, and an optional set of user interface elements that may be used to control the module's input parameters and display its output. Modules may be used programatically by passing data and parameters to the function directly, or manually by displaying the user interface to allow the user direct control over the processing behavior. Modules may be chained and nested to produce more complex analyses. User interface elements may be displayed, hidden, and recombined as needed to suit the analysis. For example, it is common for analysis modules to define a plot area as part of their user interface. When combining many such modules, it is often desirable that all modules should share the same plot area. This type of flexible recombination is supported by the analysis module architecture.

ACQ4 also provides a data-model abstraction layer which provides separation between the format of data to be analyzed and the analysis modules. This helps to ensure that data collected in different ways, or even collected by other acquisition systems, may be analyzed by the same modules as long as an appropriate data model exists to interpret each format.

#### Mosaic editor

**Figure 6:**
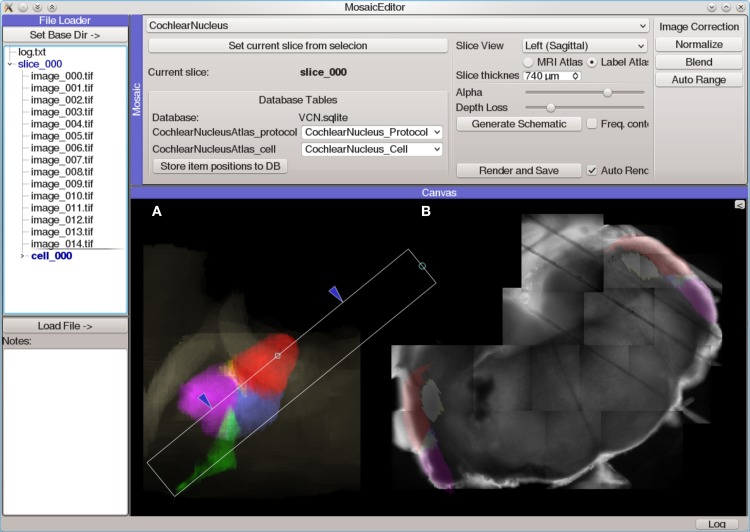
**Screen capture of the Mosaic Editor analysis module showing reconstruction and atlas registration of a mouse cochlear nucleus brain slice imaged under a 5× microscope objective**. Although the Mosaic Editor is most commonly used to simply reconstruct image mosaics, the analysis shown here is a step in registration of the data taken during the experiment to a 3D atlas of the nucleus. The images were collected with a Photometrics Quantix-57 camera and Zeiss Axioskop 2 FS microscope mounted on a manual translation stage. Positioning information was generated from a custom set of rotary optical encoders driven by the stage micrometers. **(A)** Volumetric rendering of a 3D atlas of the mouse cochlear nucleus. The white rectangle indicates the region of the nucleus from which the brain slice was taken, as determined by photos taken during the slicing procedure. This is used to create a digital slice of the 3D atlas, which is overlaid on **(B)** an automatically-reconstructed mosaic of several tiled photos of the brain slice.

The Mosaic Editor (Figure 6) provides a virtual canvas for displaying and aligning images and other data in physical coordinates. This module facilitates the creation of image mosaics which can be aligned with position-dependent data (such as photostimulation maps) and anatomical reference atlases. All data is annotated with this alignment configuration to allow further analysis to operate on normalized, corrected coordinates. An extensible atlas system provides a mechanism for creating positionable drawings and other user interface elements that allow the data to be registered with a standard atlas coordinate system. The Mosaic Editor is frequently the starting point for generating publication figures that require multiple sources of data to be aligned.

For systems which lack position feedback or for data that is otherwise misaligned, the Mosaic Editor allows the user to adjust the position and orientation of data to align it manually. These adjustments do not alter the original data, but rather are stored as meta-data alongside the original transformations. A common use case is when the sample moves unexpectedly during the experiment. In this case, data collected before the movement will be misaligned with data collected afterward. To correct this, the module allows images and other data to be drawn partially transparent, allowing the user to manually align two or more images. Once a single image is aligned correctly, the same adjustment may be immediately applied to other images so that they all share the same correction.

#### Electrophysiology analysis tools

The Event Detector module detects and measures repeated events within an analog signal. This is used primarily for detecting action potentials, synaptic currents, and calcium imaging transients. The module can be configured to make a variety of measurements on each detected event such as the amplitude and decay time constant. A table of this data is then written to a database or passed on to another module. The core analysis performed by this module is entirely customizable—a visual programming environment (similar to LabView) allows a variety of filters and detectors to be configured which determine the module's output function. This environment allows analysis routines to be rapidly prototyped and tested. It may also embed Python scripts, allowing more flexible analysis.

The IVCurve module performs basic analyses of current pulse protocols which are standard for characterizing patched cells. These analyses include computation of the resting membrane potential, cell input resistance, membrane time constant, sag in hyperpolarizing current steps, interspike intervals, first spike latency, and spike train adaptation ratios. Voltages and currents can also be plotted as a function of time for repeated stimuli, which can be useful for monitoring the effects of drug manipulations or examining plasticity of intrinsic excitability. Multiple views of the current-voltage relationship for both current and voltage clamp can be presented.

#### Photostimulation mapping tools

Although ACQ4 is designed as a general-purpose acquisition system, it has been used extensively in photostimulation mapping and thus includes a set of powerful tools for analyzing data from this type of experiment.

**Figure 7:**
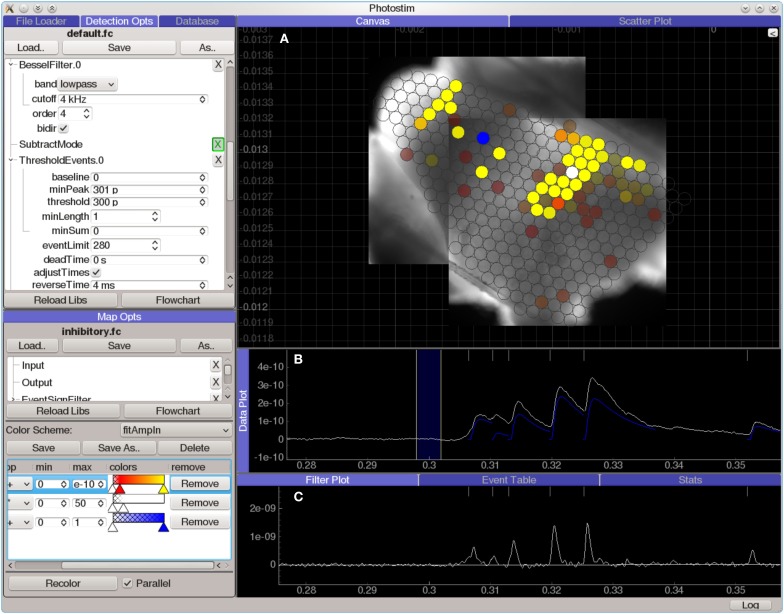
**Screen capture of the Photostim analysis module, processing laser-scanning photostimulation mapping using glutamate uncaging (300 μM MNI-glutamate) data from a mouse cochlear nucleus brain slice experiment**. The hardware used to collect the data for this experiment is described in Figure 3. A Q-switched 355 nm DPSS laser was projected through scan mirrors to uncage glutamate at locations on the slice indicated by the displayed map results. **(A)** Analyzed results from a photostimulation map overlaid on images of the brain slice used in this experiment. Brightly colored circles indicate that a strong synaptic response was detected in the patched cell when the spot was photostimulated, whereas transparent circles indicate that no response was detected. **(B)** Plot of data from a single photostimulation recording. The traces in blue indicate the amplitude and time course of the evoked synaptic currents. **(C)** A diagnostic plot showing the same data at an intermediate filtering stage in which the onset of synaptic events has been detected.

The Photostim module (Figure 7) extends the Event Detector analysis module to analyze sequences of recordings in photostimulation mapping experiments. Each recording is first analyzed to detect the onset times, amplitudes, and kinetics of PSP-like shapes. Next, the timing of events in each recording is analyzed for a variety of configurable criteria, such as the total charge transfer in a window following the stimulation, or the average decay time constant in the same window. These measurements are then used to generate colored maps, allowing spatial relationships to be visualized against images of the sample or an anatomical atlas. The Photostim module stores the results of both the event detection and the subsequent event analysis into a relational database for further analysis.

A common problem in photostimulation mapping experiments is to determine whether the synaptic events in a recording were evoked by the photostimulation or are simply part of the spontaneous activity in the cell. This is usually addressed by oversampling to determine the reliability of evoked responses, either by repeated mapping or by mapping with overlapping stimulation sites. The Map Analyzer module measures the rate of spontaneous events in a set of photostimulation recordings and estimates which stimulation sites are likely to contain evoked events. Other analysis modules provide alternate methods including a spatial correlation algorithm (Bendels et al., 2010) and simple averaging across multiple atlas-aligned maps.

#### Calcium imaging analysis

The Image Analysis module provides basic tools for analyzing imaged fluorescent indicator data combined with physiological recordings. The typical dataset for this analysis consists of a time sequence of image frames (video), optionally with intracellular or extracellular recordings of electrical activity in individual cells. The Image Analysis module provides a set of functions that support analysis of ratiometric or non-ratiometric image acquisition, frame registration for movement artifacts, time-dependent bleaching corrections, temporal and spatial filtering, and spectral calculations. Multiple regions of interest in an image may be analyzed with or without corrections, and the data exported as time series to text files for further analysis. For more complex protocols in which imaging and physiological recordings are made simultaneously, the voltage or current recordings can be displayed aligned with the image times, and spike-triggered or burst-triggered averages of data in the regions of interest can be computed.

## Conclusion and planned development

We have developed ACQ4, a complete software system for data acquisition and analysis in electrophysiology that is based entirely on free and open-source tools. Although we have placed a strong emphasis on patch-clamp electrophysiology, photostimulation, and optical imaging, ACQ4 is designed as a general-purpose acquisition system and is suitable as a platform for a wide variety of experimental paradigms in which multiple instruments must be coordinated for data collection. It is also designed to be modular, extensible, and scalable to allow the integration of new devices and experimental designs. In making this software open-source, we hope to encourage a community of developers interested in neuroscience data acquisition with Python to collaborate on ACQ4 as a centralized architecture supporting a broad range of techniques. Although ACQ4 is presently a fully functional system, several areas for future development include support for continuous acquisition, support for a broader range of devices including multi-electrode arrays and alternate data acquisition boards, integration with Neo to allow data transfer to other analysis and data sharing projects, and a larger set of acquisition and analysis modules to extend the capabilities of ACQ4.

## Authors contribution

Luke Campagnola is the primary developer of ACQ4 and author of this manuscript. Megan B. Kratz and Paul B. Manis contributed significant development to ACQ4 and changes to this manuscript.

### Conflict of interest statement

The authors declare that the research was conducted in the absence of any commercial or financial relationships that could be construed as a potential conflict of interest.
